# Extraction, Modification, Biofunctionality, and Food Applications of Chickpea *(Cicer arietinum)* Protein: An Up-to-Date Review

**DOI:** 10.3390/foods13091398

**Published:** 2024-05-01

**Authors:** Nikhil Dnyaneshwar Patil, Aarti Bains, Kandi Sridhar, Maharshi Bhaswant, Sawinder Kaur, Manikant Tripathi, Déborah Lanterbecq, Prince Chawla, Minaxi Sharma

**Affiliations:** 1 Department of Food Technology and Nutrition, Lovely Professional University, Phagwara 144411, India; nikhil2898p@gmail.com (N.D.P.);; 2 Department of Microbiology, Lovely Professional University, Phagwara 144411, India; 3 Department of Food Technology, Karpagam Academy of Higher Education Deemed to be University, Coimbatore 641021, India; 4 New Industry Creation Hatchery Center, Tohoku University, Sendai 9808579, Japan; 5 Center for Molecular and Nanomedical Sciences, Sathyabama Institute of Science and Technology, Chennai 600119, India; 6 Biotechnology Program, Dr. Rammanohar Lohia Avadh University, Ayodhya 224001, India; 7 CARAH ASBL, Rue Paul Pastur 11, 7800 Ath, Belgium

**Keywords:** chickpea, extraction, modification, food applications

## Abstract

Plant-based proteins have gained popularity in the food industry as a good protein source. Among these, chickpea protein has gained significant attention in recent times due to its high yields, high nutritional content, and health benefits. With an abundance of essential amino acids, particularly lysine, and a highly digestible indispensable amino acid score of 76 (DIAAS), chickpea protein is considered a substitute for animal proteins. However, the application of chickpea protein in food products is limited due to its poor functional properties, such as solubility, water-holding capacity, and emulsifying and gelling properties. To overcome these limitations, various modification methods, including physical, biological, chemical, and a combination of these, have been applied to enhance the functional properties of chickpea protein and expand its applications in healthy food products. Therefore, this review aims to comprehensively examine recent advances in *Cicer arietinum* (chickpea) protein extraction techniques, characterizing its properties, exploring post-modification strategies, and assessing its diverse applications in the food industry. Moreover, we reviewed the nutritional benefits and sustainability implications, along with addressing regulatory considerations. This review intends to provide insights into maximizing the potential of *Cicer arietinum* protein in diverse applications while ensuring sustainability and compliance with regulations.

## 1. Introduction

Proteins are macromolecules required for the critical functioning of cells, tissues, organs, and systems. Dietary protein intake exhibits various beneficial effects, and differently sourced (animal and plant) proteins are commonly used as food products because of their excellent functional properties [1]. Recently, plant-derived vegan proteins have attained more attention from consumers in comparison to animal-based proteins due to their prominent advantages, such as low cost, positive environmental impact, natural abundance, sustainability, and cardiovascular disease benefits [2]. In addition. due to consumers’ ever-increasing demand for plant proteins, the vegan protein market is expected to increase by 30% growing from USD 13.93 billion (2022) to USD 19.9 billion in 2023 [1]. Chickpeas belong to the Fabaceae family, and the term “chickpea” is derived from the Latin word “cicer”. It is also known by its widely used name “Garbanzo bean” [2]. Being the largest producer in the world, India produces around 70% of chickpeas in the total world production. In 2023, the world’s chickpea production was recorded at 20.5 MMT, and India contributed 13.63 MMT of chickpea production [3]. The chickpea is a valuable source of protein, carbs, fiber, ash polyphenols, flavonoids, vitamins (thiamine, niacin), minerals (Mg, Ca, Fe, P), and unsaturated fatty acids and saturated fatty acids (linoleic, oleic) [4]. The reason for the utilization of chickpeas as a food source is related to their widespread availability, substantial protein content, cost-efficiency, and excellent protein digestibility [3].

Chickpeas contain 20.9 to 25.27% of protein content, which is composed of albumin (8.39 to 12.31%), globulin (53.14 to 60.29%), gluten (3.12 to 6.89%), and prolamin (19.38 to 24.40%) [5]. Chickpea protein contains both essential and nonessential amino acids, including arginine, glutamic acid, lysine, methionine, threonine, phenylalanine, valine, tryptophan, histidine, cysteine, aspartic acid, leucine, and glycine. Plant-based proteins are an attractive alternative to animal-based proteins as their demand continues to increase because of their characteristics [6].

Choosing the precise extraction method for chickpea protein is crucial to increasing both protein yield and purity, thereby facilitating its effective use in industrial applications. Generally, wet extraction techniques (alkaline and salt extraction) are the most promising methods used to obtain purified chickpea protein with higher recovery [7]. The salt extraction (micellization) process is based on the salting-in and -out properties of proteins, which helps preserve the protein’s natural conformation without causing denaturation [8]. Alkaline extraction relies on the electrostatic repulsion between the protein molecules when the pH is increased, which exposes the hydrophobic groups and leads to protein aggregation via hydrophobic interactions. The hydrophobic groups become energized by electrostatic forces at the isoelectric point, leading to the tightening of the protein structure [8]. Comparatively, in other legumes, such as soybeans, alkaline extraction is widely used in industry for its ability to produce highly soluble and functional protein isolates [9]. Similarly, the alkaline extraction of pea proteins has shown promise as an alternative method, resulting in isolates with good solubility and emulsifying properties [10]. Lentil proteins have also been subjected to alkaline extraction for the production of functional protein isolates [11]. All wet extraction methods consume large quantities of water and chemicals, which have negative impacts on the environment. Therefore, the development of sustainable, efficient, and environmentally friendly extraction technology, such as air classification, is necessary for commercial purposes [6].

The utilization of chickpea protein in food products is hindered by its poor functional properties, including water-holding capacity, solubility, gelling, and emulsifying properties, compared to animal proteins and other legume proteins. To mitigate these limitations, various modification methods, including physical modification, biological modification, and chemical modification, have been employed to enhance the functional properties and biological activities of chickpea proteins [7]. The physical modification involves altering the protein structure through the application of a force field, which enhances its functionality, digestibility, and biological activities. For instance, chickpea proteins exhibit improved solubility and stability after undergoing ultrasonication treatment, making them more suitable for use in beverage products [12]. Biological modification involves the alteration of the functional properties and biological activities of proteins by breaking down or constructing protein structures. For example, chickpea proteins show improved foaming and emulsifying properties after undergoing enzymatic hydrolysis [13]. Chemical modification involves the reaction of proteins with chemical reagents to change their structures by breaking and forming chemical bonds, thereby enhancing their functionality. For instance, chickpea protein solubility, emulsifying stability index (ESI), and emulsifying activity index (EAI) are improved by acetylation with acetic anhydride, making chickpea proteins more suitable for utilization in the food industries for various applications [14].

Chickpea protein is often used in food industries as a valuable ingredient for producing a variety of plant-based dairy products, including yogurt, milk, tofu, beverages, and sattu. It is also used in cereal-based products, pasta, gluten-free noodles and muffins, and sausages, where it serves as a meat analog due to its functional properties, such as gelling, emulsifying, and foaming [15]. Based on various modifications of chickpea proteins, it can also be used in novel areas, such as microencapsulation and protein-based films, due to the improved solubility, gelation, and rheological properties [16].

This study pioneers the detailed analysis of chickpea protein, focusing on extraction and modification methods and their effects on functional properties. Unlike previous reviews on plant proteins, this work delves into the less-explored chickpea protein, uncovering innovative insights into extraction optimization and new modification techniques. It fills a significant gap in the scientific literature, showcasing chickpea protein’s potential in various applications and laying a crucial foundation for future research. This represents a significant advancement in plant-based protein science.

## 2. Extraction Technique of Chickpea Protein

The extraction of chickpea protein is a crucial step in determining its quality and potential applications. Various methods are used for this purpose, including wet milling, dry fractionation, alkaline extraction, ultrafiltration, and salt extraction. Each method has its pros and cons, such as cost, protein yield, and quality. Wet milling involves soaking chickpea seeds in water to extract high-quality proteins with a high yield for food and nutraceutical applications [17]. Dry fraction extraction, in contrast, does not require water and separates protein from chickpea seeds mechanically, yielding a high-protein fraction for diverse food applications [18]. Alkaline extraction employs sodium hydroxide (NaOH) to dissolve chickpea protein, enhancing its solubility, functionality, and nutritional value. This approach finds widespread application within the food industry for a variety of legume seeds, including chickpeas [18]. Since no literature is available on the extraction of chickpea protein using green extraction techniques, further research is needed on green protein extraction techniques, such as ultrasound-assisted extraction, supercritical fluid extraction, pulsed electric field, and microwave-assisted extraction.

### 2.1. Wet Milling

**Figure 1:**
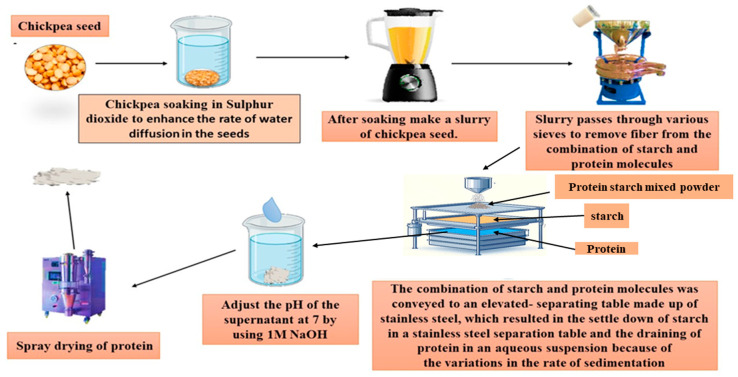
Chickpea protein extraction using wet extraction technique.

Wet milling is a process used to extract proteins from biological materials, such as plant seeds, animal tissues, and microorganisms. The process involves the use of high-speed stirring or homogenization to mechanically break down the tissue, followed by various separation and purification steps to isolate the desired protein. This industrial-level process involves the use of automated diaphasic centrifuges, hydro-cyclone, and sieves [19]. Chickpea seeds are first soaked in sulfur dioxide, which increases the rate of water diffusion, reduces the interaction between protein and starch by breaking disulfide bonds, and inhibits microbial growth [17]. After soaking the seeds, they are ground in the slurry, and this resulting mixture is then filtered through various sieves to separate the fibers (Figure 1). The remaining slurry (starch and protein) is transferred to a stainless-steel separation table, where protein is drained in an aqueous suspension and starch settles due to differences in sedimentation rates [20]. The suspension containing protein further undergoes centrifugation, neutralization, drying, and defatting before being obtained as the final product (Figure 1) [21]. Giteru et al., [22] show that wet milling has several advantages, including high protein purity (94.2%), yield (49%), and reduced fat content, but also has limitations, such as high water and energy consumption, high waste control costs, and difficulty in handling and potential loss of functional properties. The use of sodium bisulfite enhances the solubility of the protein; however, it causes a small decline in certain amino acids, including aspartic acid, glutamate, leucine, lysine, cysteine, phenylalanine, arginine, and tryptophan (Table 1). Furthermore, the study by Espinosa-Ramírez et al. [23] compares the effect of wet milling and isoelectric precipitation extraction techniques on chickpea protein functionality. The investigation revealed that the wet milling extraction of chickpea protein (WMCP) displayed low protein yield (11.26%), reduced fat content (94.36%), and enhanced fat absorption capacity (37.33%) compared to isoelectric precipitation (IPCP). These are attributed to the mechanical breakdown process of wet milling, which effectively separates protein from other components, such as fat, resulting in improved purity and reduced fat content while also facilitating better fat absorption capacity [15]. Remarkably, WMCP had negligible effects on protein content, solubility, emulsifying, and foaming capacities compared to IPCP. However, WMCP exhibited low water-holding capacity (139.10%) and foaming stability (37.88%). This can be attributed to the mechanical processing involved in wet milling, which may disrupt the natural protein structure, leading to decreased functionality in terms of water retention and foam stability [24]. Additionally, WMCP displayed slightly lower in vitro protein digestibility (2.79%) and amino acid content (21.05%), which may result from the mechanical shearing forces applied during wet milling, which could potentially alter the protein’s tertiary and quaternary structures, impacting its digestibility and amino acid composition [25]. Examination through SDS-PAGE revealed a loss of protein fractions due to wet milling, while secondary structures remained unaltered based on ATR-FTIR spectra analysis.

### 2.2. Dry Fraction

Dry fractionation, shown by methods like milling and air classification, offers an environmentally sustainable approach to protein extraction from leguminous sources [26]. This process involves finely milling materials to release starch and fragment protein bodies, facilitating the separation of larger starch granules or fiber-heavy particles from smaller protein-rich components [27]. Dry fractionation conserves water by eliminating the need for it in the process and preserves the functionality of extracted elements [28]. Despite its advantages, protein concentrates from dry fractionation may exhibit lower purity compared to wet extraction isolates and could contain inherent anti-nutrient elements [29]. Tribo-electrostatic separation and air classification are notable techniques within dry fractionation [28]. Overall, dry fractionation presents a viable method for sustainable protein extraction, with specific considerations regarding purity and potential anti-nutrient content. 

#### Air Classification

**Figure 2:**
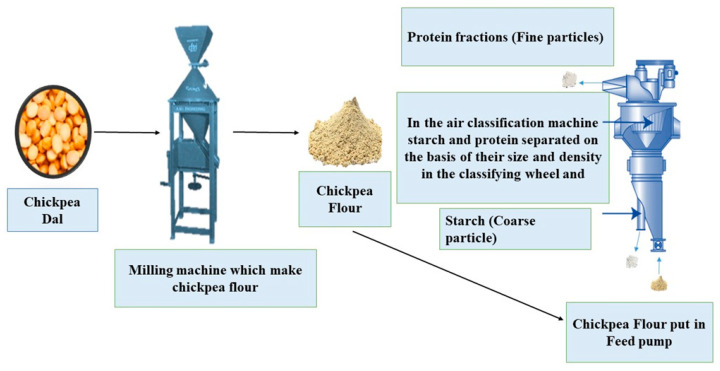
Chickpea protein extraction using air classification method.

The fundamental principle that forms the basis of air classification is the tendency of particles within an airstream to separate according to their size and density. In this process, milled flour is introduced into a separation chamber and subjected to fluidization using airflow [30]. The dominant methods for fractionating flour involve impact and jet milling, complemented by rotor-type classifiers (Figure 2). Air classification has demonstrated its effectiveness in extracting protein from leguminous sources such as peas, fava beans, and lupin. In certain cases, a preceding initial dehulling stage may become essential to remove particular anti-nutrient elements, enhance the color of protein flour, reduce astringency or bitterness, and potentially boost the protein content [18]. In the initial stages, finely milled flour, having traversed an airstream, enters a classifier wheel in rotation. Within this region, the milled flour within the bed undergoes centrifugal separation, leading to the formation of smaller protein-rich fragments measuring around 3μm and larger starch granules spanning from 25 to 40μm. This culminates in the creation of a protein-rich fraction or concentrates [31]. The design of the classifier wheel is intentionally tailored to enable the precise separation of smaller particles while excluding larger ones. The larger particles gather in the lower section of the chamber, forming the coarse starch fraction [32]. The coarse fraction is distinguished by its elevated starch and fiber content, whereas the fine fraction is abundant in protein. As an option, the coarse fraction can undergo re-milling and be reintroduced into the air classifier, which further enhances the purity and yield of each fraction (Figure 2). This process hinges on two critical factors: the velocity of the air classifier and the milling speed, both of which are of paramount importance [24]. Adequate milling speed is imperative to guarantee comprehensive cell disruption, which assists in the segregation of starch and protein fractions. Nevertheless, it is crucial to acknowledge that one drawback of the air classifier method is its relatively reduced protein yield when compared to wet extraction techniques [18]. The protein yield in concentrates, obtained through air classification from diverse pulse-based sources, has been documented to range from 49% to 70% [33]. The investigations led by Assatory et al. [34] and Xing et al. [35] reported chickpea protein extraction with 29–46% protein content and 11–31% purity. This method shows lower energy consumption and a reduced environmental impact compared to wet milling for protein extraction. By primarily utilizing air movement, it requires less energy input and water usage, minimizing its environmental footprint [31]. Chickpea protein shows excellent foam formation, emulsification, and solubility. However, it has drawbacks, such as low yield, high fat content, powder fouling, and anti-nutritional factors [36]. Air classification yields lower protein purity compared to wet milling extraction due to differences in protein separation mechanisms. Wet milling solubilizes and recovers proteins more effectively, while air classification relies on physical principles and may not fully extract proteins bound to other components [17]. However, recycling the stream in air classification can increase extraction yield by further separating protein particles from the feed material [35]. This process provides additional opportunities for capturing more protein, which is particularly useful for fine particles or when significant protein remains in the coarse fraction [37]. Additionally, air classification may not disrupt cellular structures as efficiently, leading to protein loss and lower overall yield [18]. The study by Xing et al. [35] shows that optimal extraction in air classification relies heavily on the speed of the classifier wheel. Speeds ranging from 6000 to 8000 rpm ensure a balance between separation efficiency and particle integrity. However, higher speeds, around 10,000 rpm, can lead to increased yield by enhancing the separation process.

### 2.3. Wet Extraction Methods 

Wet extraction methods utilize aqueous solvents like acids or alkalis, along with water, to extract proteins from raw materials by solubilizing them for subsequent recovery through precipitation or separation. The incorporation of solvents can significantly enhance protein recovery, particularly when combined with other techniques [38]. Starting materials for extraction may include milled particles, flakes, or air-classified protein fractions. Drying these materials is often necessary to improve shelf stability, especially during transportation, as it reduces moisture content and prevents microbial growth, enzymatic degradation, and chemical reactions that could compromise the quality and stability of the protein extracts [25].

#### 2.3.1. Alkaline Extraction Followed by Isoelectric Precipitation

**Figure 3:**
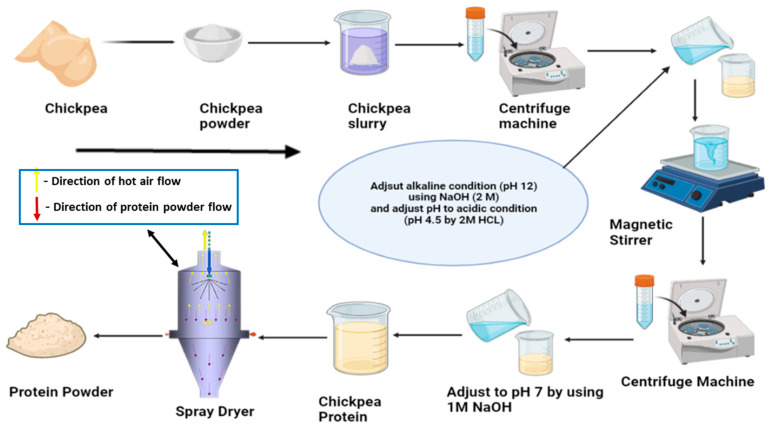
Chickpea protein extraction using alkaline extraction followed by isoelectric precipitation.

The conventional procedure for protein extraction from botanical specimens typically involves alkaline solvents, commonly potassium hydroxide or sodium hydroxide, to adjust the pH of the extraction solution. The alkaline conditions, typically within the range of 8 to 11, disrupt disulfide bonds within proteins, enhancing their solubility and facilitating their release and retrieval [18,39]. Protein precipitation is then initiated by introducing an acid, such as hydrochloric acid, to lower the pH, causing proteins to reach their isoelectric point and precipitate. The subsequent separation of insoluble constituents, primarily fibers and starch, from the settled protein fraction, can be achieved through centrifugation, filtration, or sieving techniques [40]. Optionally, a purification step involving rinsing with water or an acidic solution, followed by re-dissolving at a neutral pH with an alkaline solution, may be incorporated to enhance protein quality. Heating is often employed for microbiological safety before dehydration, typically via freeze-drying in laboratory settings or spray-drying in industrial-scale operations [41]. However, spray drying is associated with limitations, such as a 50% recovery rate and denatured protein functionality due to the intricate bonds formed between polysaccharides and solvents within the cellular matrix, hindering the release of proteins [42]. Despite the apparent purity of proteins used, spray drying involves a complex mixture containing polysaccharides and other components from the original source material (Figure 3). During drying, interactions between these components and solvents can occur, forming bonds that reduce functionality. This reduction is influenced not only by the purity of proteins but also by the overall composition of the mixture [42]. Alkali application for protein release can lead to amino acid conversion, reduced digestibility, and the degradation of essential amino acids like lysine and cysteine [18]. Nonetheless, the optimization of parameters, including alkaline concentration, sample-to-solvent ratio, temperature, and extraction duration, can mitigate challenges and enhance protein yield while maintaining cost-effectiveness [43]. Alkaline extraction is favored for its elevated digestibility and the bioavailability of isolated proteins [44]. In an investigation conducted by Chang et al. [5,45,46], chickpea protein was efficiently obtained through alkaline extraction and subsequently precipitated at its isoelectric point, which is at a pH of 4.5. This method consistently yielded elevated protein yields ranging from 67% to 85%, coupled with remarkable protein purity levels spanning from 68% to 85% (Table 1). In another study conducted by Zhang et al. [47], the chickpea protein concentrate extracted through alkaline extraction shows a protein yield of 60.69%. The protein has satisfactory functional attributes and amino acid content, except sulfur-contacting amino acids. Comparative studies show that alkaline protein extraction typically yields high protein content and purity compared to air classification due to the solubilization of proteins under alkaline conditions, leading to efficient extraction. Alkaline solvents like potassium hydroxide or sodium hydroxide disrupt protein–protein and protein–cell matrix interactions, facilitating the release of proteins from botanical specimens. Consequently, the extracted proteins tend to have higher purity levels because they are less contaminated by other cellular components [5,45,46]. However, compared to wet milling extraction, alkaline protein extraction may have lower protein content and purity. Wet milling methods involve the mechanical or chemical disruption of plant material, which can lead to a more comprehensive extraction of proteins and other desirable components. Additionally, wet extraction techniques often involve multiple purification steps, such as filtration or centrifugation, which further enhance protein purity [25].

#### 2.3.2. Ultrafiltration Technique

**Figure 4:**
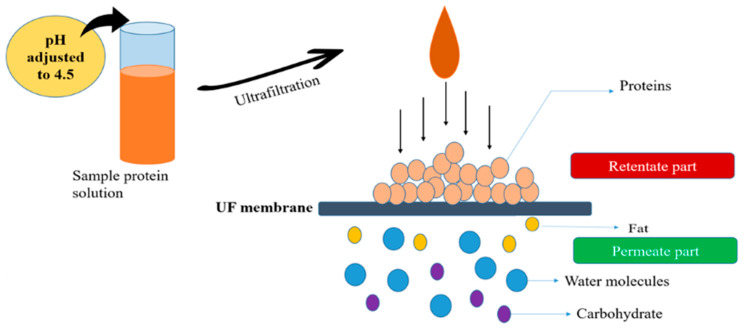
Chickpea protein extraction using ultrafiltration technique.

Ultrafiltration (UF) is a method employing a selectively semipermeable membrane to separate dissolved proteins. The protein solution is directed through ultrafiltration membranes featuring pores that specifically retain proteins while enabling smaller soluble components like carbohydrates to pass through (Figure 4). Following this, the isolated protein can be subjected to either spray-drying or freeze-drying, mirroring the process used for isolates obtained through isoelectric precipitation [48,49]. Ultrafiltration presents numerous potential advantages compared to the isoelectric precipitation technique utilized in acid and alkali extraction processes. These benefits encompass the ability to retain a broader spectrum of protein constituents from the extract, including albumins, as opposed to acid and alkali methods, which frequently prioritize the retrieval of globulins [50]. Furthermore, due to the elimination of the need for extreme pH conditions in this process, proteins obtained through ultrafiltration (UF) may retain their native conformation. Additionally, the omission of a neutralization step leads to isolates with decreased levels of sodium and ash content but an augmented yield of protein [18]. Regarding the functional attributes of protein derived from lentils, peas, and chickpeas that have undergone processing through isoelectric precipitation and ultrafiltration techniques [51]. The research indicated that the highest protein content and purity were achieved through ultrafiltration (UF) at pH levels of 9 and 6, compared to UF at a pH of 9 and diafiltration (DF) at a pH of 9. DF at a pH of 6 and UF at a pH of 9 resulted in lower phosphorus levels, while DF at a pH of 9 and UF at a pH of 9 led to reduced phenol content. However, both methods had high trypsin inhibitor levels [52,53,54].

#### 2.3.3. Use of Enzymes in Protein Extraction

Enzymatic-assisted extraction provides a solution to numerous challenges linked to both acid and alkali-assisted techniques for protein extraction. Enzymes play a pivotal role in facilitating protein liberation by enzymatically breaking down cell wall polymers and improving protein solubility to a certain degree [55]. As a result, enzyme-assisted extraction presents a viable alternative to alkaline extraction due to its environmentally friendly nature and reduced negative impacts. Prominent examples of extracting enzymes encompass proteases (such as alcalase) and carbohydrases (including cellulose and pectinases) [56]. When comparing enzyme-assisted extraction to acid extraction, enzymes, particularly proteases, are favored as pretreatment agents for alkaline extraction. Proteases enhance protein yield by breaking the bonds between proteins and the polysaccharide matrix. Additionally, they partition higher molecular weight proteins into more soluble forms, thereby enhancing the effectiveness of the extraction process [57]. Moreover, proteases function within their optimal pH range, preventing the denaturation of extracted proteins. Additionally, they hinder the creation of complexes between unconventional proteins and various cellular elements, such as polysaccharides [58]. The proteins obtained through this method exhibited resistance to oxidation and had low viscosities, signifying their suitability for incorporation into food products. However, it has some limitations, which encompass its complexity, operational expenses, difficulties in upscaling the procedure, inconsistent protein retrieval, and substantial energy consumption [59]. However, despite these challenges, the enzymatic protein extraction technique is advantageous because of its minimal impact on the environment and its positive impact on protein functionality and recovery [46]. As per the research conducted by Perovic et al. [46], the enzyme-aided alkaline extraction of protein from chickpeas yielded a remarkable 30-fold increase in extracted protein yield, achieving a 93% protein recovery rate. Additionally, this process resulted in enhanced functional properties, including a 14% increase in solubility, a 130.54% increase in water-holding capacity, a 22.31% increase in oil absorption capacity, and a remarkable 150.26% improvement in emulsifying ability and foaming properties.

#### 2.3.4. Salt Extraction

Salt extraction is the technique used for legume protein extraction in which salt is added to the protein mixture, which helps to solubilize the protein and separate it from other components. The salt extraction technique is based on the salt in and out mechanism [60]. Salting-in refers to the phenomenon in which the addition of salt increases the solubility of proteins in aqueous solutions. This is because the salt can interfere with the hydrophobic interactions that hold the protein molecules in a stable conformation, causing the protein to become more soluble. Conversely, salting-out refers to the opposite phenomenon, in which the addition of salts reduces the solubility of proteins in aqueous solutions. This is because salt can interfere with the electrostatic and hydrophobic interactions that hold the protein molecules in a stable conformation, causing the aggregation of protein and becoming less soluble [61]. In the salt extraction process, the legume protein mixture is subjected to a high concentration of salt, typically in the form of NaCl or KCl. The salt will denature the protein, causing it to become more soluble and separate from other components, such as carbohydrates and fats. This is due to the interference of the salt with the hydrophobic interactions that hold the protein molecules in a stable conformation [62]. The protein-rich fraction can then be separated from the other components through methods such as centrifugation, filtration, or sedimentation. The resulting protein can then be washed and further processed to increase the protein content. The salt extraction process provides several benefits, including reduced processing time and cost, improved product yield, and increased protein solubility, which can be useful for applications such as functional foods and beverages [63]. The effectiveness of salt extraction in extracting legume proteins can be affected by various parameters, including the pH of the solution, initial protein content, type of legume, and salt concentration [16]. The study by Karaca et al. [64] indicates that the protein yields obtained using salt extraction of lentil, fava bean, chickpea, and pea protein are 74.71, 81.98, 81.63, 81.09, and 82.64%, which is less than the protein yield given by the alkaline extraction technique. This study also shows that the protein extracted using the salt extraction technique has lower solubility than the alkaline extracted protein.

**Table 1:** Extraction methods of chickpea protein.

Method	Protein Yield (%)	Protein Purity (%)	Advantages	References
Wet milling	11.26–49	49.36–94.2	High protein yield, fat absorption rate, and starch particle integrity. Light in color Low content of fat	[5,19,44]
Air classification and milling	11–31	29–46	Less energy requirement Preserve the native functionality of a protein. Chemical free treatment Higher water-holding capacity, foaming capacity, emulsification, and nitrogen solubility index.	[32]
Alkaline extraction followed by isoelectric precipitation	85–90	86–91	high-yield protein high protein purity	[5,45]
Ultrafiltration	67–87	50–55	High product throughput Lower complexity Commercial availability of systems with high-productivity designs Prevents loss of enzyme activity Minimal product degradation Protein retention increases with time	[52,53,54]
Salt extraction	80–90	88–90	Increasing hydration and water binding capacity Increasing the binding properties of proteins to improve texture Reducing solvent consumption	[64]
Enzymatic extraction of protein	93	80–95	Increases the protein yield and purity Enhanced the functional properties	[46]

## 3. Techno-Functional Properties of Chickpea Protein

### 3.1. Protein Solubility (PS)

The protein’s solubility is an important factor that is affected by various factors like temperature, ionic strength, pH, and the type of solvent used. Research on the chickpea protein’s solubility showed that it has minimum solubility at a pH of 4 to 5, which is the isoelectric point of the protein, and maximum solubility at a pH of 1 to 3 and 7 to 10 [65,66]. This is because, at the isoelectric point, the protein has a net zero charge, and thus, the ionic hydration and electrostatic repulsion between the protein molecules decrease, leading to precipitation [67]. On the other hand, when the pH is higher or lower than the isoelectric point, the interaction between the protein and water increases due to the negative or positive charge of the protein [68]. The studies have shown that the solubility of protein extracted using isoelectric precipitation (91.20%) is higher compared to wet extraction (67.3%), salt extraction (30.16%), and ultrafiltration (60%) [60,69,70,71]. The difference in solubility among various protein extraction techniques stems from their distinct mechanisms. Isoelectric precipitation achieves high solubility by maintaining proteins in their native form, minimizing denaturation or aggregation [68]. In contrast, wet extraction methods often involve harsh conditions, leading to protein denaturation and reduced solubility. Salt extraction may cause protein aggregation due to excessive salt concentration and competition with protein–water interactions [68]. Ultrafiltration, while effective in removing impurities, can also lead to the loss of smaller proteins that are crucial for solubility and may induce conformational changes or aggregation [68].

### 3.2. Oil-Holding Capacity (OHC)

The oil-holding capacity of protein is an important parameter in the food industry, affecting the organoleptic and structural qualities of food [60]. The protein–lipid interaction is driven by multiple factors, such as electrostatic repulsion, hydrophobic groups, hydrogen bonds, and noncovalent bonds [72]. The oil-holding capacity of protein powder can be influenced by particle size and density; smaller, less dense particles generally have higher oil-absorption capacities compared to larger, denser particles [73]. A study found that Kabuli chickpea protein has an OHC range between 2.29 and 4.16 g/g, which is higher than the Desi variety of chickpea protein (2.21 to 3.69 g/g), due to its higher percentage of non-polar amino acids [74]. This suggests that incorporating protein in a concentrated or isolated form in the diet could lead to better fat retention [74]. It was also observed that protein extracted through wet extraction (2.33 g/g) had a higher oil-holding capacity than protein extracted through isoelectric precipitation (2.30 g/g) [60,69,70,71]. The difference in oil-holding capacity between protein extracted through wet extraction (2.33 g/g) and isoelectric precipitation (2.30 g/g) may stem from varying degrees of protein denaturation [72]. Wet extraction methods can induce more denaturation, potentially enhancing oil binding, despite protein unfolding [74]. In contrast, isoelectric precipitation maintains protein structure, leading to a slightly lower oil-holding capacity. This highlights the influence of extraction techniques on protein functionality [74].

### 3.3. Water-Holding Capacity (WHC)

The water absorption capacity of a protein refers to its capacity to hold water against gravitational forces through various physiochemical interactions [75]. The protein’s conformation and structure play a role in its ability to bind water molecules via hydrogen bonds with hydrophilic groups, such as carboxyl, carbonyl, amino, sulfhydryl, and hydroxyl groups, on the side chains of proteins [76]. The water-holding capacity of a protein impacts its potential food applications, as it affects the organoleptic and structural qualities of food compositions containing the protein [77]. A high water-holding capacity can result in a dry and brittle texture, especially when stored for extended periods, while a low water-holding capacity results in low water retention efficiency [60]. Chickpea protein isolates and flour have water-holding capacities of 2.41 to 3.48 g/g and 1.7 g/g, respectively, with isolates having a larger capacity due to more binding sites being revealed through swelling, separation, unfolding, and exposure [78]. Defatted chickpea protein concentrate (3.0 to 3.6 g/g) has a higher water-absorption capacity compared to non-defeated chickpea protein (2.3 to 2.9 g/g), highlighting a notable impact of processing on the functional attributes of protein constituents [79,80]. Studies have shown that proteins extracted through isoelectric precipitation (3.65 g/g) have a higher water-holding capacity than proteins extracted through wet extraction (1.56 g/g), dry extraction (1.2 g/g), and ultrafiltration (2.7 g/g) techniques [60,69,70,71]. This difference in water-holding capacity can be attributed to the preservation of protein structure during isoelectric precipitation, which minimizes denaturation [60]. In contrast, other extraction methods may induce protein denaturation, resulting in a reduced water-binding ability [76].

### 3.4. Emulsifying Capacity and Stability (EC and ES)

The emulsification ability of dietary proteins is shaped by their emulsifying efficacy. This efficacy, in turn, is dependent on a combination of internal and external factors. Internal factors include the properties of the protein itself, such as the charge, polarity, solubility, molecular weight, structure, and conformational stability, and external factors, such as pH, ionic strength, and temperature, also play a role in emulsion stability by affecting the interaction between the protein and the emulsion components. [81]. Proteins in an emulsion system form a coating over oil droplets, which are suspended in an aqueous medium, thus preventing undesirable phenomena such as flocculation, creaming, coalescence, and sedimentation [60]. The emulsifying activity index (EAI) evaluates a protein’s ability to emulsify oil, measuring the maximum interfacial area per gram of protein in a stable emulsion. Emulsifying capacity (EC) is defined as the amount of oil emulsified per gram of protein [82]. The emulsifying stability (ES) is used to measure an emulsion’s ability to resist structural modifications with respect to time [83]. The study revealed that the Kabuli variety of chickpea protein had a greater EAI (1.6 to 1.9 min) and EC (26.47 to 28.5 m^2^/gm) than the Desi variety (1.2 to 1.5 min and 23.85 to 25.11 m^2^/gm, respectively) [84]. The EC of a protein also depends on the extraction method used. Chickpea protein isolate (CPI) exhibits an emulsifying capacity of 70–80% and an emulsifying stability of 60–70% [60]. In comparison, pea protein isolates have a wider range of emulsifying activities (21–76%), with variable stability. White lentil and cowpea protein isolates show high emulsifying activity (68–69%) but may have similar stability to CPI [85]. Fava bean protein isolate (FPI) can range from 24% to 85% in emulsifying capacity, with varying stability [86]. Soybean protein isolate generally has higher emulsifying stability compared to CPI and other legume proteins [87]. According to several studies, protein extracted via isoelectric precipitation had a lower EAI (25.17 m^2^/gm) and ESI (14.09 min) than protein extracted via salt extraction (38.83 m^2^/gm and 10.92 min, respectively) [60,69,70,71].

### 3.5. Foaming Capacity

Proteins can stabilize foams due to their tendency to adsorb on the air–water interface and interact with other proteins to lower surface tension and create durable membranes. Foam stability and capacity indices provide an evaluation of foam qualities [88]. Foam capacity refers to the increase in volume achieved by whipping the protein solution, while the stability of foam is measured by the variation of foam volume over a certain time period. The foaming ability of a protein is largely determined by its cohesive, flexible, elastic, and interfacial film properties, which allow it to bind and retain air [89]. A study found that different varieties of chickpea protein had a foaming capacity ranging from 33 to 47% [75]. An additional study demonstrated that when the foaming capacity of chickpea protein concentrate is expressed as the ratio of foam volume after 1 min to the liquid volume before whipping, it ranges between 25 and 47% (Table 2) [75]. The investigation revealed that chickpea flour can produce a low-volume foam that is moderately stable and has a foaming capacity of more than 91.76% after 2 h of storage. This implies that the natural proteins found in chickpea flour exhibit good water solubility and demonstrate strong surface activity [90]. The foaming capability of various varieties of chickpea protein ranges from 36.9 to 41% [23]. Several studies show that chickpea protein obtained through isoelectric precipitation has a lower foaming capacity of around 62%, compared to protein obtained through ultrafiltration, which had a foaming capacity of about 90%. However, protein extracted through isoelectric precipitation showed a higher foam stability of 94.49% compared to protein extracted using the dry extraction method, which had a foam stability of 89.2%. These findings suggest that the choice of protein extraction method can significantly impact the foaming properties of chickpea protein [60,69,70,71].

### 3.6. Gelling Properties 

The formation of gels from dietary proteins, particularly globular proteins, occurs when these proteins are denatured by heating in an aqueous solution and the interaction between the protein and solvent reaches a balance [91]. Globulins exhibit the ability to dissociate and re-associate during heating, leading to the production of different gels with varying characteristics [92]. Partially unfolded proteins can also transform into gels by forming crosslinked networks of uncoiled polypeptide fragments. The gel-forming capability of a protein is determined by its minimum gelling concentration, or the low protein concentration required to develop a gel structure [93]. Research has shown that the lowest gelling formation concentration of chickpea protein ranges from 16 to 20%, higher than that of chickpea flour, which ranges from 8 to 12% [94]. A study found that the Kabuli variety of chickpeas developed a strong gel-like structure at a concentration of 14%, while the Desi variety required a concentration of 17% [95]. This difference in gel-forming concentration can be attributed to variations in protein ingredients and non-protein constituents, such as carbohydrates and lipids. The formation of gel is greatly influenced by parameters such as protein content, heat treatment, ionic/reducing agents, non-protein constituents, and pH conditions [10]. A study indicated that the formation of gel in chickpea protein isolate obtained through wet extraction, ultrafiltration, and isoelectric precipitation occurred at concentrations ranging from 4.5 to 11.5%, suggesting that the protein extraction method greatly impacts gel formation [96].

**Table 2:** Functional properties of chickpea protein.

Functional Property	Description	Influencing Factors	Extraction Method Impact	References
Protein Solubility (PS)	Minimum solubility at pH 4 to 5 (isoelectric point), maximum at pH 1 to 3, and 7 to 10. Higher solubility with isoelectric precipitation (91.20%) compared to other methods	pH, temperature, ionic strength, solvent type	Isoelectric precipitation achieves higher solubility (91.20%), while wet extraction (67.3%), salt extraction (30.16%), and ultrafiltration (60%) may lead to denaturation and lower solubility.	[60,69,70,71]
Oil-Holding Capacity (OHC)	Influenced by particle size, density, and amino acid composition Kabuli chickpea protein has higher OHC than the Desi variety Wet extraction can result in higher OHC	Particle size, density, amino acid composition, extraction method	Wet extraction may induce protein denaturation leading to higher OHC (2.33 g/g), while isoelectric precipitation maintains protein structure, resulting in slightly lower OHC (2.30 g/g).	[60,69,70,71]
Water-Holding Capacity (WHC)	Isolates have higher WHC than flour; defatted concentrate has higher WHC than non-defatted. Higher WHC with isoelectric precipitation compared to other methods	Protein conformation, extraction method	Isoelectric precipitation maintains protein structure, resulting in higher WHC (3.65 g/g), while other methods may induce denaturation and lower WHC (1.56 g/g for wet extraction).	[60,69,70,71]
Emulsifying Capacity (EC)	EC is higher in the Kabuli variety compared to the Desi variety Wet extraction results in higher EC compared to isoelectric precipitation	Protein properties, pH, ionic strength, temperature	Wet extraction may induce more denaturation, enhancing EC (26.47 to 28.5 m²/g), while isoelectric precipitation maintains protein structure, resulting in slightly lower EC (25.17 m²/g).	[60,69,70,71]
Emulsifying Stability (ES)	Influenced by protein properties, pH, ionic strength, and temperature The Kabuli variety has a higher ES than the Desi variety	Protein properties, pH, ionic strength, temperature	The extraction method impacts ES, with wet extraction generally having higher stability compared to isoelectric precipitation (e.g., 14.09 min for isoelectric precipitation).	[60,69,70,71]
Foaming Capacity	Chickpea protein foaming capacity ranges from 33 to 47% Isoelectric precipitation results in lower foaming capacity but higher stability compared to ultrafiltration	Protein properties, pH, temperature, extraction method	Ultrafiltration may result in higher foaming capacity (around 90%), but isoelectric precipitation leads to higher stability (around 94.49%).	[60,69,70,71]
Gelling Properties	The Kabuli variety forms gel at lower concentrations than the Desi variety The extraction method affects gel formation concentration	Protein content, heat treatment, ionic/reducing agents, non-protein constituents, pH	Gel formation concentration is influenced by the extraction method, with wet extraction requiring lower concentrations compared to isoelectric precipitation (e.g., 4.5 to 11.5%).	[60,69,70,71]

## 4. Modification of Chickpea Protein 

### 4.1. Physical Modification

The physical post-modification of protein involves the application of external forces that result in alterations of a protein structure, either individually or as part of the food matrix. This method typically leads to unfolding, disaggregation, redistribution, and reduction of size, or permanent denaturation of the protein [97]. Because limited literature is available on the physical post-modification of chickpea protein (ultrasonication and high pressure), further research is needed on the techniques, such as heat treatment, cold plasma technology, and Gamma irradiation to evaluate the effects on the functional and nutritional properties of chickpea protein.

#### 4.1.1. Ultrasonication Technique

**Figure 5:**
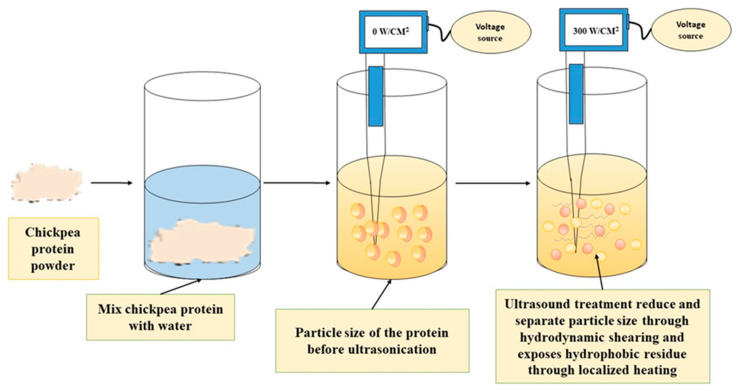
Chickpea protein modification using ultrasonication.

Ultrasonication is a novel technology for the modification of plant-based protein at low cost and proper functionality [98]. Ultrasonication technology involves the transmission of sound waves above 16 kHz, beyond the range of human hearing (Figure 5). These waves cause a longitudinal displacement in the medium, resulting in alternations of rarefaction and compression [99]. This technology is divided into two frequency ranges, including high-frequency ultrasonication (ranging from 100 kHz to 1 MHz, with a power less than 1 W/cm^2^), which is commonly used to analyze the physical and chemical properties of food, and low-frequency ultrasonication (16 kHz to 100 kHz, with power ranging from 10 to 1000 W/cm^2^), which is primarily used to modify proteins physically and chemically (Figure 5) [100,101]. The effects of ultrasonication are related to cavitation-induced heating, dynamic agitation, shear stresses, and turbulence, which affect the functional characteristics and biological functions of the resulting protein constituents [102]. 

The functionality of proteins is improved through ultrasonication treatment by reducing the particle size of protein molecules. This reduction is achieved via turbulence, cavitation, and shear force generated by the ultrasonication process in the solution [103]. The breakdown of unstable protein aggregates into smaller particles increases the collision and aggregation rate caused by these forces [104]. Furthermore, it has been observed that ultrasonic treatment imparts energy that breaks non-covalent connections, such as hydrogen bonds and hydrophobic interactions, between proteins, leading to a reduction in the particle size of protein [105].

A study showed that ultrasonication treatment (150 W for 30 min) increased the solubility and water-holding capacity (WHC) of chickpea protein by 26.6% and 38%, as compared to non-treated protein (Table 2). This improvement was attributed to the utilization of cavitation and mechanical effects of ultrasonication to disintegrate larger protein aggregates into smaller protein particles, thus reducing protein size and enhancing protein–water interaction and solubility [106]. Additionally, it was observed that ultrasonic treatment can partially denature protein molecules and increase the exposure of hydrophilic groups to water, leading to improved WHC of protein (Figure 5).

The ultrasonication treatment (300 W for 20 min) increased the gelling properties of chickpeas protein by 157.8% as compared to native protein [80]. The formation of a gel-like structure in a protein solution occurs due to the interaction between attractive and repulsive forces between protein molecules during heating [107]. The texture of the gel is regulated by the breaking force, which is impacted by ultrasonic time. An increase in ultrasonic time results in an increase in breaking force and vice versa [108]. When a protein solution is heated to form a gel, it can transform free sulfhydryl groups into disulfide bonds. Hence, a higher concentration of free sulfhydryl in protein leads to the formation of additional disulfide bonds, producing a more stable gel structure with increasing breaking force [109].

Studies have shown that ultrasonication treatment (300 W for 20 min) increased the emulsifying and foaming capacity of chickpea protein by 8.3% and 136.7% as compared to non-treated protein [80]. This improvement is attributed to the exposure of hydrophobic groups within chickpea protein after ultrasonication treatment, resulting in more flexible protein molecules and enhanced emulsifying and foaming properties [110]. The physical modification of chickpea protein using ultrasonication shows high potential in improving its functional properties for various food applications.

#### 4.1.2. High-Pressure Treatment (HP)

High static pressures, commonly falling within the range of 200 to 700 MPa, have found extensive application in the alteration of specific plant proteins [111]. This modification procedure, referred to as high-pressure processing (HPP), is influenced by different conditions, including the treatment duration, temperature, magnitude of applied pressure, and characteristics of the protein solution, such as pH and ionic strength [112]. These elements collectively induce structural alterations that impact the functionality of the protein, particularly its gelation properties. The process by which protein modification occurs through high-pressure treatment is fundamentally based on its ability to initiate alterations in the volume of protein molecules when they are in a liquid state [113]. This leads to the rupture of protein molecules and their denaturation and subsequent aggregation. This phenomenon is rooted in Le Chatelier’s principle, which establishes an inverse relationship between pressure and volume. An increase in pressure promotes a decrease in volume, and conversely, a decrease in pressure results in an increase in volume [114]. In a solution, a protein’s overall volume encompasses the space taken up by its atoms, the volume of any empty spaces or pockets resulting from imperfect folding, and the volume alteration that occurs due to its interaction with the solvent in the solution [115]. Hence, employing high pressure leads to the compression of protein voids, the disruption of non-covalent interactions (which includes intramolecular hydrophobic and electrostatic interactions), and the formation of novel non-covalent or semi-covalent connections [116]. It is important to highlight that hydrogen bonds are notably affected only at exceedingly high pressures, typically at or above 1000 MPa. [117]. High-pressure conditions do not substantially compromise the stability of covalent bonds in proteins, thereby upholding their primary structural configuration. Nevertheless, heightened pressure does exert a discernible influence on the secondary, tertiary, and quaternary levels of protein structure [118]. It is important to highlight that the impact of high-pressure processing (HPP) on the functional properties of proteins displays variability dependent on the particular attributes inherent to the plant-based protein being examined [119]. In a study by Ma et al. [120], varying homogenization pressures and cycles were applied to chickpea protein. This process enhanced surface hydrophobicity, decreased sulfhydryl content, and reduced particle size while maintaining molecular weight. These modifications improved solubility, foaming, and emulsifying properties, resulting in more stable emulsions as compared to native protein. Furthermore, in the study by Alsalman et al. [121], a central composite rotatable design was utilized, with varying pressure levels (227–573 MPa), treatment times (6–24 min), and chickpea protein powder concentrations (11–29%). High-pressure (HP) treatment notably enhanced emulsion properties compared to untreated samples. Protein aggregates decreased by 33.3%, β-sheets showed reductions ranging from 4.2% to 87.6%, and α-helix content dropped by 50%, resulting in improved protein digestibility as compared to native protein. While some treated samples exhibited intensity changes, the overall band trends remained stable. HP treatment also reduced thermal denaturation temperature (Td) and enthalpy due to increased denaturation, signifying its influence on protein structure and functionality. In the study by Huang et al. [122], chickpea protein was subjected to high-pressure processing (HPH) at 90 MPa, resulting in notable functional improvements. Apparent viscosity increased from 185.69 to 521.16 Pas, with enhanced frequency dependence and protein recovery rate (42.80% to 54.27%) and improved protein solubility, at 0.448%, compared to native protein. Additionally, particle diameter significantly decreased, reaching 0.677 μm with 90 MPa treatment and 1.89 μm with 150 MPa treatment as compared to non-treated protein. These findings underscore HPH as a promising approach to enhancing chickpea protein functionality.

### 4.2. Chemical Modification 

The chemical modification of proteins involves the alteration of their native structure by inducing chemical reactions that break or form new covalent bonds. The chemical reactions target specific protein side chains, such as the indole imidazole, thioester, disulfide/sulfhydryl, carboxyl, phenolic, and amino groups, thereby modifying the biophysical and functional properties of the protein [123]. Additionally, chemical modification can also modulate the protein’s net charge by replacing the other hydroxyl/amino or ε-amino group of particular amino acids. As a result of these modifications, chemically modified proteins typically have enhanced functionality associated with unmodified protein fragments [124]. Because limited literature is available on the chemical post-modification of chickpea protein (acetylation), further research is needed on the techniques, such as acylation, glycosylation, deamination, cationization, and phosphorylation, to evaluate the effects on the functional and nutritional attributes of chickpea protein.

#### 4.2.1. Acetylation 

Acetylation is a chemical modification process that involves the addition of an acetyl group to the hydroxyl group of an amino acid residue in a protein. This modification is commonly used to improve the functional characteristics of the protein. In the process of acetylation, acetic anhydride is commonly utilized as the acetylation agent due to its high reactivity, good solubility, and low steric hindrance properties compared to other acetylation agents, such as tetrahydrofuran-tetra carboxylic anhydride and benztri carboxylic anhydride [125].

**Figure 6:**
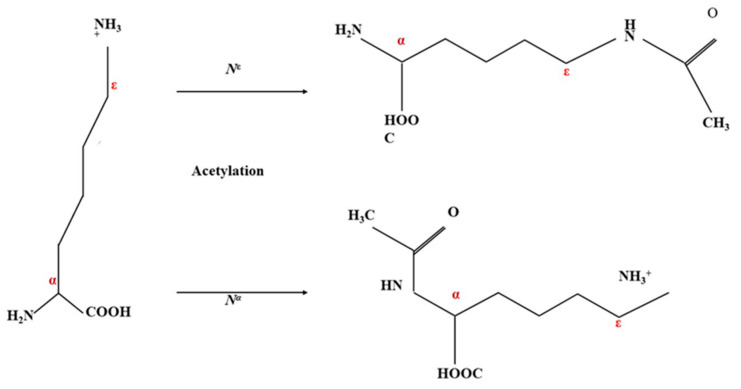
Chickpea protein modification using acetylation.

The addition of the acetyl group to the protein results in a decrease in the protein’s net surface charge and a decrease in the molecular weight of the protein, leading to the unfolding of the polypeptide chain (Figure 6). The acetylation process also increases the balance of aromatic and aliphatic residues without changing the amino acid profile of the protein, except for lysine [126]. Hydrogen bonds are of paramount importance in regulating protein–water interactions, and these interactions are contingent upon the protein’s electronegativity. The process of acetylation renders the protein more electronegative, consequently promoting the creation of additional hydrogen bonds with the surrounding medium. As a result, this enhanced interaction enhances the protein’s solubility [127].

This study showed that the solubility of the protein increased after acetylation treatment by 52% at a pH of 7.5–10. During the acetylation, acetyl groups are covalently added to the protein’s amino group, which causes a certain amount of protein unfolding (reduced electrostatic attraction); because of this, hydrophilic groups of the protein are exposed, which leads to a rise in hydrophilicity and enhances the protein solubility [128]. When the pH of the solution is 2–7, the solubility of acetylated proteins may decrease due to the formation of hydrophobic linkages between alkyl and aromatic groups of amino acids. These linkages can lead to protein aggregation, which can be stronger than the number of hydrophilic cation groups present, resulting in low solubility. The aggregation of proteins due to hydrophobic interactions can reduce their solubility and impair their functional activity [129].

A study showed that the emulsifying capacity of chickpea protein increased by 19% after acetylation because it promotes the formation of lipophobic and hydrophobic residues, which enhances the protein’s ability to act as an emulsifying agent [128]. Acetylation also enhances the foaming ability of protein as it decreases the positive charges of the ε-amino groups of lysine, resulting in reduced molecular size of the protein and enhancing the interaction between water and air [130]. The chickpea protein was treated with 2.5% (W/V) acetic anhydride and maintained a pH of 7.5–8, leading to degrees of acetylation of 6 and 49% [131]. The chemical modification of chickpea protein using acetylation shows high potential in improving its functional properties for various food applications. 

#### 4.2.2. Succinylation

Succinylation is a protein modification process wherein succinic anhydride reacts with protein amino groups, forming succinylated proteins. This chemical modification introduces succinyl groups (-COCH_2_COOH) onto the protein backbone, altering its physicochemical properties and functionalities. The mechanism of succinylation involves the nucleophilic attack of the primary amino group (-NH_2_) of the protein’s lysine residues on the carbonyl group of succinic anhydride. This results in the formation of an unstable intermediate, which then undergoes intramolecular rearrangement to yield a stable succinylated protein and a molecule of succinic acid. The succinylation process can occur under various conditions, typically at a mildly alkaline pH to facilitate the reaction between the amino groups and succinic anhydride. Additionally, the reaction may require the presence of a catalyst, such as triethylamine, to enhance the reaction rate. In the study by Patil et al. [132], the succinylation modification of chickpea protein increases its functional attributes, like water-holding capacity, oil-holding capacity, solubility, and emulsifying capacity by 39.83, 54.02, 7.20, and 23.17%. This increase in functional attributes is due to succinylation-induced modifications, such as changes in surface properties and protein structure. 

### 4.3. Biological Modification

#### 4.3.1. Enzymatic Modification

Enzymatic modification entails utilizing both proteolytic and non-proteolytic enzymes to alter the structural features of a protein. Depending on the desired outcome, this method can be applied to either degrade or enhance a protein’s structure, thereby achieving the desired functionality [133]. In enzymatic modification, non-proteolytic enzymes like transglutaminases (TG) are employed to enhance the structural integrity of proteins. and textural properties by protein-cross linking, while proteolytic enzymes such as alcalase, pepsin, papain, and trypsin are used to break down the peptide linkage of the protein’s primary amino acid sequence, thereby changing its functional properties during hydrolysis [134]. Enzymatic modification is a widely used technique in the field of protein chemistry and biotechnology to improve the functional properties of proteins [135].

In an enzymatic modification, proteins are hydrolyzed by enzymes, which results in the breakage of peptide bonds and the separation of the substrate into short-chain peptides and amino acids with lower molecular weights [136]. The effectiveness of the enzymatic hydrolysis process relies on multiple factors, such as the optimal pH and temperature of the substrate and enzyme, the type of protein substrate and enzyme, the process parameters (pressure, temperature, and pH), the enzyme-to-substrate volume ratio, and the absence or presence of proteolytic inhibitors. To achieve improved functionality or higher yields, it is crucial to conduct the hydrolysis procedure under optimal conditions. Ensuring that the pH is adjusted to the ideal level is particularly important to prevent enzyme inactivation during the process [137].

##### Enzymatic Modification Using Alcalase and Flavourzyme

**Figure 7:**
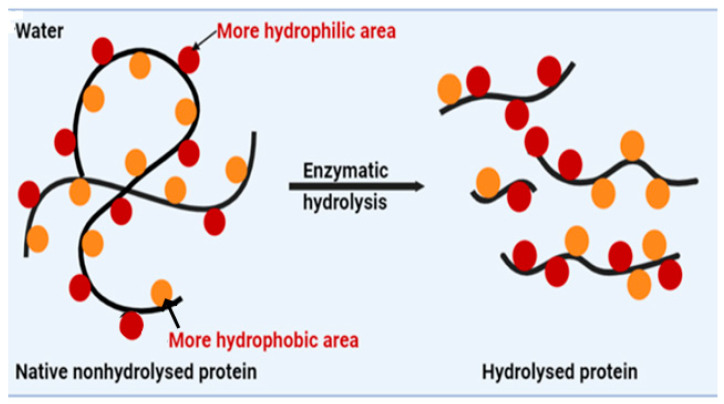
Chickpea protein modification using enzymatic hydrolysis.

Two of the most used enzymes for protein hydrolysis are flavourzyme and alcalase. Flavourzyme, a mixture of endoprotease and exopeptidase derived from *Aspergillus oryzae*, is primarily used to cleave amino acids from the end of polypeptide chains during protein hydrolysis (Figure 7) [138]. Alcalase, an endopeptidase derived from *Bacillus licheniformis*, has a wide activity catalytic and can break the peptide bonds in the interior of polypeptide chains [139].

A study showed that after the “enzymatic hydrolysis using alcalase”, protein content was increased by 6.64%, with an intensification in the degree of hydrolysis, which shows that the insoluble protein fraction is gradually reduced as a result of the proteolytic breakage of peptide bonds, while the yield of soluble protein increases [140,141]. In another study, the solubility of modified protein increased by 70% when the pH was more than 7 [142]. This improvement of protein solubility during alcalase enzymatic hydrolysis is due to the reduction in molecular weight and an increase in soluble peptides from insoluble aggregates or precipitates, as well as a concurrent rise in ionizable amino and carboxyl groups [143]. 

The emulsifying capacity of hydrolyzed chickpea protein varies based on the degree of hydrolysis. At a 4% degree of hydrolysis, the emulsifying capacity increased by 20% due to increased solubility, molecular flexibility, and exposure to hydrophobic regions of the polypeptides (Figure 7). This is because a lower degree of hydrolysis results in larger protein molecules, which occupy a larger surface area and promote molecular rearrangement and adsorption, leading to improved emulsifying capacity [144]. However, at a 14.67% degree of hydrolysis, the emulsifying capacity decreased by 44% because, at high degrees of hydrolysis, the densely packed protein molecules at the interface prevent full unfolding or reorientation [145].

Another study on chickpea protein hydrolysis using alcalase showed that there was a decrease in interfacial tension by 10.67% (Table 2). This is attributed to the release of small peptides into the solution, which diffuses into the air–water interface and competes with one another, leading to a reduction in the rate of diffusion. The change in conformation and molecular size of the protein due to enzymatic hydrolysis influences the rate of diffusion of these peptides to the interface [146]. In “enzymatic hydrolysis using Flavourzyme”, the protein content was decreased by 1.22%, 2.5%, 2.55%, 3.6%, and 5.07% at the degrees of hydrolysis 1, 3, 5, and 10%, respectively [142] (Table 2). This investigation reveals that hydrolysis using flavorzyme improved the solubility of protein at all pH levels, except for a degree of hydrolysis of 1% [147]. The greatest difference in solubility between chickpea protein and its hydrolysates was found at the isoelectric point. An increase in solubility was observed at a pH range of 4 to 6 as the degree of hydrolysis increased, particularly at 3–5% [148]. These results are consistent with the modification of chickpea protein using the alcalase enzyme. However, chickpea protein hydrolysis at 3% produced with flavorzyme showed lower solubility at an acidic pH level than chickpea protein hydrolysis at 3% produced with alcalase. Hence, the selectivity of proteases and the degree of hydrolysis play a critical role in increasing solubility [142].

The ability of chickpea proteins to absorb oil was increased by 109.67, 114.21, 63.32, and 58.74% as a result of enzymatic hydrolysis at 1, 3, 5, and 10%, respectively [149]. This may be because protein hydrolysis exposes non-polar side chains that bind hydrocarbon moieties of oil and increase oil absorption. Due to the significant exposure of ionic groups, the hydrolysate with DH 3% had the highest oil absorption ability, and as the degree of hydrolysis increased, the absorption capacity decreased [150]. 

Other important functional properties are emulsifying activity and stability. The results showed that in hydrolysis at 1% and 3%, there was an increase in the emulsifying activity of chickpea protein by exposing hydrophobic amino acid residues that interact with oil [151]. However, the smaller peptides produced from hydrolysis were less stable compared to whole proteins and led to decreased emulsion stability [152]. The flavorzyme-glyoxyl derivative had increased the emulsifying activity by 18% but decreased stability by 8.10% at 1% degree of hydrolysis. The 3%, 5%, and 10% degrees of hydrolysis also negatively affect the stability, i.e., it decreased by 6.9, 10.96, and 4.02%, respectively (Table 2).

The foaming capacity of chickpea protein was improved by 126% at a degree of hydrolysis of 1% (Table 2). Additionally, higher degrees of hydrolysis resulted in further improvement in foaming capacity, with the highest stability observed at a degree of hydrolysis of 3%, where the foam was able to retain 60% of its structure after 60 min of exposure to ambient temperature [153]. The conditions for enzymatic modification of chickpea protein using alcalase and flavourzyme involved adding chickpea protein in water at a 1:1 ratio at 80 °C for 5 min. The pH was then maintained at 8, and the temperature was held at 50 °C [143].

##### Enzymatic Crosslinking Using Transglutaminase Enzyme

Transglutaminase is an enzyme used to crosslink dietary proteins to improve the strength of the polypeptide structures and enhance the textural qualities of the protein [154]. This enzyme stands out in its uniqueness as it is the sole food-grade crosslinking enzyme accessible for commercial use. Transglutaminase operates through the facilitation of acyl transfer reactions and the formation of crosslinks between lysine residues, which serve as acyl acceptors, and glutamine residues, which serve as acyl donors, either within or between protein chains [155]. The efficiency of transglutaminase for crosslinking was studied using chickpea protein solutions, with optimal results observed at a pH of range 5 to 8. This enzyme is stable within a broad pH range [156]. 

**Figure 8:**
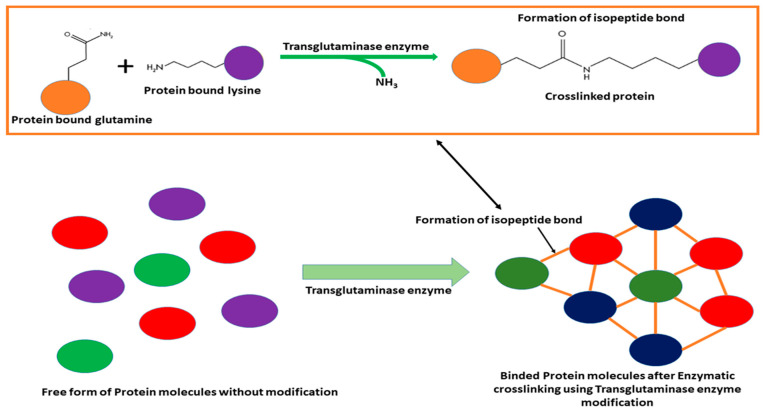
Chickpea protein modification using transglutaminase enzyme.

The stability of the emulsion generated by the electrostatic attraction between negatively charged proteins depends on the high zeta potential of the protein’s dispersion during emulsification [157]. The addition of transglutaminase to the chickpea protein stabilized the emulsion, resulting in an increase in zeta potential by 14% [156] (Table 3). This increase in zeta potential led to an improvement in emulsion stability as the oil droplets covered with chickpea protein had a greater net charge, which was sufficient to counter various attraction forces like van der Waals, hydrophobic, depletion, and stabilized repulsive electrostatic forces between oil droplets [158]. The addition of transglutaminase also resulted in a shift in particle size toward larger particles, i.e., more than 100 µm, due to the aggregated particles, leading to the development of a gel-like structure in the emulsion [159]. The non-crosslinked emulsion remained largely unchanged in the presence of 1% SDS, but it showed a reduction in average diameter size, indicating coagulation primarily formed by hydrophobic, hydrogen, and electrostatic interactions (Figure 8). This development of a stable particle system resulted in the crosslinked emulsion having a gel-like structure [160].

In vitro, gastrointestinal simulation studies have shown that the addition of transglutaminase decreases the digestibility of chickpea protein. This decrease in digestibility has been attributed to the formation of isopeptide bonds in the transglutaminase crosslinked proteins [161]. However, toward the end of the gastric phase, the crosslinked chickpea emulsion became more digestible due to the breakdown of the crosslinks and the increased availability of enzymatic cutting points for pepsin, which facilitated hydrolysis [159]. In vitro digestion studies have confirmed that the chemical composition of the crosslinks also contributes to the lower digestibility of the crosslinked material [162].

**Table 3:** Modification techniques of chickpea protein.

Type of Method	Treatment Condition	Functional Changes	Reference
Ultrasonication	Frequency at 20 kHz Power 300 W (on time 4 s, off time 2 s) For 5, 10, and 20 min.	Improved protein solubility (26.6%) Increase emulsifying capacity (8.3%) Increased foaming capacity (136.7%) Improved water-holding capacity (38%) Improved gelling properties. (157.8%)	[76]
High-pressure treatment	Pressure 90–150 MPa	Apparent viscosity increased from 185.69 to 521.16 Pa s, Improved protein solubility at 0.448%. Particle diameter significantly decreased up to 0.677 μm with 90 MPa treatment and 1.89 μm with 150 MPa treatment	[116]
Acetylation	Degree of Acetylation (6–49%) Acetic anhydride (2.5% W/V) 2N NaOH at pH 7.5–8	Solubility increased at pH 7.5–10 (52%). Solubility decreased at pH 2.0–7.0. Improved water absorption capacity (45%) Increase oil absorption capacities (64%). Increase emulsifying capacity (19%).	[125]
Hydrolysis	Hydrolysis with Flavourzyme (1–10% DH) Temperature 4–50 °C (1 h) pH 7 (100 mM NaH_2_PO_4_)	Enhanced solubility of protein at DH 3 and 5% Decrease solubility at DH 1% Decreased protein content (2.5, 2.55, 3.6, and 5.07%) at DH 1, 3, 5, and 10%. Improved oil absorption capacity (109.67, 114.21, 63.32, and 58.74%) at DH 1, 3, 5 and 10%. Increase foaming capacity (126%) at DH 1%. Reduced emulsifying activity (6.9, 10.96, and 4.02%) at DH 3, 5 and 10%. Increased emulsifying activity (18%) at DH 1%	[143]
	Hydrolysis with Alcalase (4–15% DH) pH 8.0 (4N NaOH) Temperature 50–80 °C Time 20–210 min	Increased protein solubility (70%) at pH 7. Low solubility at pH 4–5. Increased protein content (6.64%) up to DH 8.6%. Decreased interfacial tension (10.67%). Decreased emulsifying capacity (44%) at DH 14.67%. Increased emulsifying capacity (20%) at DH 4%.	[144]
Crosslinking With transglutaminase	Hydroxamate assay TG used 200 U/per g of protein Temperature 37 °C Time 6 h.	Development of a gel-like emulsion, Enhanced emulsion stability Decrease protein digestibility	[163]

## 5. Application of Chickpea Protein: Food Industry

**Figure 9:**
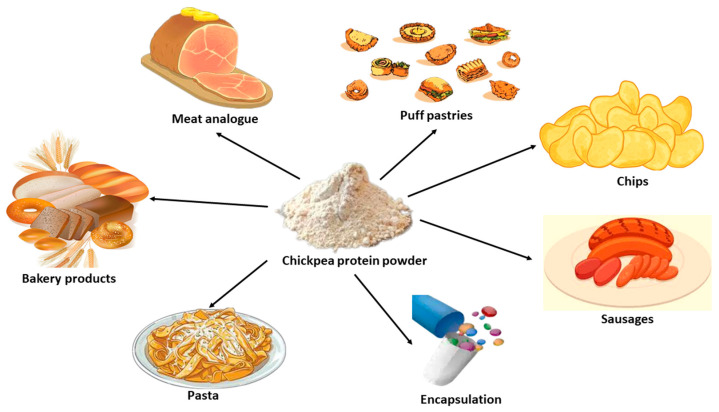
Application of chickpea protein in various food products.

Chickpea protein fractions serve as a versatile and sustainable protein source in a variety of food applications, including meat analogs, infant formula, and cereal-based and bakery products (Figure 9). The addition of chickpea protein enhances the total protein content while preserving the quality of cereal-based foods in terms of both nutrition and sensory attributes [164]. Additionally, the partial replacement of wheat flour with chickpea flour in food products, such as bread, pasta, and baked goods, leads to an increase in protein content and an enhanced nutritional composition, as well as improved rheological, functional, and sensory attributes [165,166]. Chickpea protein has been utilized in the formulation and development of pasta products with lower glycemic indices. These pasta products are currently available on the market and are manufactured by various multinational food companies. [167]. Scientific studies have shown that the utilization of chickpea flour in pasta preparation results in a significant reduction in the glycemic response, as measured by the rate of sugar release into the bloodstream [94]. A study has demonstrated that incorporating chickpea flour in baked goods results in a decrease in their in vitro glycemic response, providing an opportunity for the creation of food items with low glycemic indices [168]. Furthermore, the incorporation of protein-rich chickpea flour in the formulation of puff pastries and chips, in conjunction with other components, has been reported [161]. A research study has determined that the addition of chickpea flour and protein concentrate, at concentrations of 1.5%, 2.5%, and 5%, to cooked sausage significantly enhances the sensory characteristics of merguez sausage [169]. The utilization of chickpea protein concentrates in meat-based products has been demonstrated to improve color stability during storage, reduce lipid oxidation, and enhance antioxidant capacity. [170]. A study has examined the effect of replacing a portion of wheat flour with chickpea protein concentrates in bread production. The results indicated that the bulk bread volume made with 6% and 9% chickpea protein was greater than that of the control bread made entirely with wheat flour, although the magnitude of the increase was less pronounced compared to the control [171]. The authors documented that the reduced water vapor production observed during bread baking could be responsible for the elevated water absorption capacity of plant-based proteins [172]. A study was conducted to examine the addition of Kabuli and Desi chickpea proteins to newborn formula, which increases the nutritional standards set by the WHO in terms of carbohydrate content, protein composition, amino acids, and most micronutrients, with only minor supplementation of vitamins, minerals, and oil (Table 4). To minimize the concentration of anti-nutritional components, initial treatments, such as enzymatic hydrolysis, dehulling, germination, and boiling, were utilized [173]. The study demonstrated that chickpea protein components exhibit strong emulsifying and foaming stability, as well as good gelling properties, and can therefore be effectively used as a soy substitute in the production of sausage-like meat substitutes [174]. In the market, there is an increasing number of yogurt substitutes made with chickpea protein concentrate. Additionally, the nutraceutical industry utilizes chickpea protein isolates as a means of encapsulating micronutrients, as demonstrated in a study in which folate was encapsulated using chickpea protein isolates [75]. The use of protein for micronutrient encapsulation is a growing technology with significant potential due to its biocompatibility and nutritional significance [75]. The incorporation of chickpea protein in biscuits increases the nutritional values and textural properties of the biscuits [175]. The addition of chickpea protein in mayonnaise enhances the textural properties, aroma, flavor, appearance, and aroma, which increases the acceptance rate of the mayonnaise in the market [176]. The incorporation of chickpea protein in wheat flour muffins increases the viscoelasticity, decreases the crust hardness, reduces the browning index, and increases the sensory attributes of the muffins [177].

## 6. Conclusions

Chickpea protein presents a promising alternative to animal-based proteins due to its numerous functional and nutritional benefits. With its high protein content and diverse amino acid profile, chickpea protein offers the potential for various food applications. However, its utilization is hindered by poor functional properties compared to animal proteins, such as water and oil-holding capacity, emulsification, foaming, and gelling. These properties are influenced by factors such as cultivars, extraction methods, and modification techniques. Various extraction methods, including wet milling, dry fractionation, alkaline extraction, ultrafiltration, and salt extraction, are used to extract chickpea protein for food and nutraceutical applications. While these methods offer benefits in yield and quality, there is a lack of literature on green extraction techniques for chickpea protein. Among extraction methods, alkaline extraction, followed by isoelectric precipitation, is considered one of the best, yielding high-quality protein with minimal contamination. In modification techniques, various approaches are employed to enhance the functional properties of chickpea protein. Physical methods, such as ultrasonication and high-pressure treatment, along with chemical techniques like acetylation and succinylation, offer promising avenues for improvement. Additionally, biological methods, such as enzymatic hydrolysis and transglutaminase crosslinking, also show potential. Physical modification emerges as one of the best approaches. Techniques such as heat treatment, ultrasonication, and high-pressure processing improve the functional properties of chickpea protein without compromising its nutritional integrity, as compared to other methods. These methods enhance solubility, water, and oil-holding capacity, as well as emulsification and gelling properties, making chickpea protein more suitable for various food applications. Chickpea protein demonstrates remarkable versatility and sustainability in various food applications, contributing to enhanced nutritional profiles, improved sensory attributes, and functional benefits in a range of products, including meat analogs, baked goods, pasta, and infant formula. Its utilization offers opportunities for developing healthier and more appealing food options with lower glycemic indices and enhanced protein content. Additionally, chickpea protein’s potential in encapsulating micronutrients and substituting for soy in meat substitutes highlights its growing importance in both the food and nutraceutical industries. As research continues, future directions should focus on exploring green extraction techniques, such as ultrasound and supercritical fluid extraction, for chickpea protein. Additionally, novel modification methods, including heat treatment, cold plasma, and gamma irradiation, as well as chemical modifications like acylation and glycosylation, hold promise for enhancing protein functionality in various food applications. However, more investigation is needed to understand taste, nutrition, and digestibility in meat analogs and their integration into dairy and flour products without affecting flavor and texture. Feasibility in 3D-printed food also requires further study.

## Figures and Tables

**Table 4:** Application of chickpea protein in food products.

Food Products	Application	Reference
Cereal-based food product	Enhance nutritional value Enhance organoleptic properties	[164]
Meat Product	Increase organoleptic properties, Maintain color during storage Reduce lipid oxidation	[174,170]
Capsule and Micronutrient supplementation	Increase the biocompatibility Nutritional advantage Improve loading capacity Reduce toxicity Improve stability of folate vitamin B9.	[75]
Gluten-free muffins	Decrease the crust hardness Decrease browning index Enhanced viscoelasticity	[177]
Gluten-free noodles and pasta	Enhanced the protein content Increase antioxidant properties Decrease the glycemic index Decrease starch digestibility	[167]
Soy-Wheat bread	Increase the hardness and Chewiness Decrease the lower specific loaf volume	[147]
Yogurt	Enhanced the growth of probiotic bacteria Increased antioxidant capacity and viscosity	[75]
Mayonnaise	Enhance the acceptability in terms of texture, aroma, flavor, appearance, aroma	[176]
Biscuit	Enhance the nutritious values low digestibility Improve textural properties	[175]
Snacks	Increases the nutritional quality Improve physical properties Enhanced sensory qualities Increased storage stability	[166]
Merguez Sausage	Increase process yield Improve textural and sensorial properties	[169]

## Data Availability

The original contributions presented in the study are included in the article, further inquiries can be directed to the corresponding authors.
